# Small Ruminant Piroplasmosis: High Prevalence of *Babesia aktasi* n. sp. in Goats in Türkiye

**DOI:** 10.3390/pathogens12040514

**Published:** 2023-03-26

**Authors:** Mehmet Can Ulucesme, Sezayi Ozubek, Aleyna Karoglu, Zeliha Irem Turk, Irem Olmus, Bunyamin Irehan, Munir Aktas

**Affiliations:** Department of Parasitology, Faculty of Veterinary Medicine, University of Firat, Elazig 23200, Turkey

**Keywords:** *Babesia aktasi* n. sp., small ruminant, PCR-RLB, prevalence

## Abstract

Small ruminant piroplasmosis is the hemoparasitic infection of sheep and goats caused by *Babesia* and *Theileria* species responsible for clinical infections with high mortality outcomes. The disease is transmitted by ixodid ticks and prevalent in the tropical and subtropical regions of the world, including Türkiye. A prevalence survey, using molecular methods, is conducted in this study to determine the frequency of newly defined *Babesia aktasi* n. sp. and other tick-borne piroplasm species in small ruminants in Turkiye. A total of 640 blood samples from sheep (n = 137) and goats (n = 503) were analyzed by nested PCR-based reverse line blot (RLB) hybridization. The results show that 32.3% (207/640) of apparently healthy, small ruminants are infected with three *Theileria* and two *Babesia* species. *Babesia aktasi* n. sp. was the most prevalent species in goats, with 22.5% of samples being positive, followed by *B. ovis* (4%), *T. ovis* (2.8%), *T. annulata* (2.6%), and *Theileria* sp. (0.6%). None of the sheep samples were positive for *Babesia aktasi* n. sp.; however, 51.8% were infected with *T. ovis*. In conclusion, the findings reveal that *B. aktasi* n. sp. is highly prevalent in goats, but absent in sheep. In future studies, experimental infections will determine whether *B. aktasi* n. sp. is infectious to sheep, as well as its pathogenicity in small ruminants.

## 1. Introduction

Tick-borne diseases caused by protozoa, such as babesiosis and theileriosis, are a major economic burden on both domestic and wild animals worldwide [1,2,3]. Babesiosis, caused by the *Babesia* species, is prevalent in tropical and subtropical regions, and is considered a zoonotic disease [2,4]. *Babesia* species that are known to infect sheep and goats are *Babesia ovis*, *B. motasi*, *B. crassa*, *B. taylori*, and *B. foliate* [1]. *Babesia ovis* has been known to cause clinical babesiosis; consequently, the disease has a significant economic impact on small ruminant breeding [5]. The main vector for *B. ovis* is *Rhipicephalus bursa*, and the primary clinical signs of the disease are a fever, anemia, jaundice, and hemoglobinuria [6,7]. *Babesia motasi*, which is transmitted by *Haemaphysalis* spp. ticks, has a variable pathogenicity, with high pathogenicity in the Mediterranean Basin and low pathogenicity in Northern Europe [8]. *Babesia crassa*, a species that has low pathogenicity in goats, has been reported in Iran; however, the vector of the parasite remains unknown. [1]. Additionally, sporadic cases of human babesiosis caused by *B. motasi* and *B. crassa* have recently been reported in Asia [9,10].

The use of molecular diagnostic techniques to investigate *Babesia* species has led to the discovery of new *Babesia* species. Several *Babesia* isolates, named *B. motasi*-like (*Babesia* sp. BQ1 (Lintan), *Babesia* sp. BQ1 (Ningxian), *Babesia* sp. Tianzhu, *Babesia* sp. Madang, *Babesia* sp. Hebei, and *Babesia* sp. Liaoning), have been reported in small ruminants in China [11,12]. Based on the genome analysis of *Babesia* sp. Xinjiang, a parasite that infects sheep in China and is transmitted by *Hyalomma anatolicum* and *Haemaphysalis quinghaiensis* ticks, it has been determined that it is a species separate to the *B. motasi*-like group [11,13,14]. *Babesia venatorum*, a zoonotic pathogen infecting deer in Europe, has been found in sheep in the UK; however, it is still unclear whether sheep are also a reservoir [15].

Small ruminants have been observed to be infected by various *Theileria* species and genotypes, some of which cause extremely lethal infections [16,17]. *Theileria lestoquardi*, *T. luwenshuni*, and *T. uilenbergi* are known to cause malignant theileriosis, whereas *T. ovis*, *T. recondita*, and *T. seperata* are known to cause non-pathogenic, benign theileriosis [18]. *Theileria* sp. OT1 was isolated from sheep in Spain, and *18S rRNA* gene-based phylogenetic analysis revealed that it was identical to *T. luwenshuni* [19]. *Theileria* sp. OT3 and *Theileria* sp. MK have also been identified in sheep and goats. The pathogenicity and vectors of these three genotypes (*Theileria* sp. OT1, *Theileria* sp. OT3, and *Theileria* sp. MK) are unknown [19,20,21].

Reverse line blot (RLB) hybridization, an effective and practical diagnostic tool for simultaneously detecting tick-borne bacteria and protozoans using specific oligonucleotide probes, has also enabled the discovery of many genotypes and species previously unknown in *Theileria* and *Babesia* genera [22,23]. In our recent molecular survey, conducted in goats from the Mediterranean region of Türkiye, a novel *Babesia* sp. clearly different to ovine *Babesia* species described to date was reported by PCR-based RLB [23]. In a subsequent study, this new *Babesia* sp. (*Babesia aktasi* n. sp.) was isolated from a naturally infected goat, and the genetic and morphological characterizations of the parasite were assessed [24]. However, the frequency and distribution of *B. aktasi* n. sp. in goats has not been established in the region. There is also no information on whether *B. aktasi* n. sp. is present in sheep. In the resent study, we perform a nested PCR-based RLB survey on apparently healthy, small ruminants in the Mediterranean region of Türkiye. The study’s goals are to determine the prevalence and distribution of *B. aktasi* n. sp. in small ruminants, as well as other *Babesia* and *Theileria* species in the region.

## 2. Materials and Methods

### 2.1. Study Region, Collection of Blood Samples, and DNA Extraction

**Figure 1:**
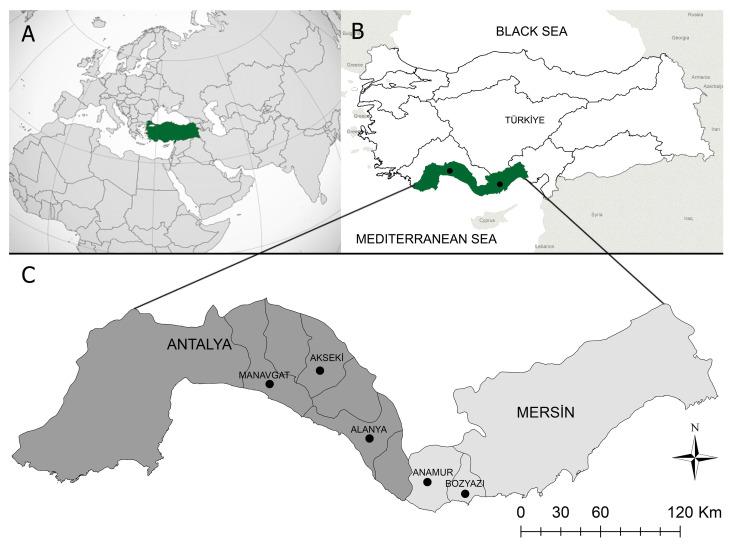
Map of Turkiye, illustrating the sample-collection region. (**A**):Turkiye’s placement on the globe map. (**B**): The area of the study, which took place in Turkiye’s Mediterranean region. (**C**): The study area included two provinces and five local districts.Blood samples from 640 apparently healthy, small ruminants (503 goats, 137 sheep) were collected into vacuum tubes containing anticoagulant (K3-EDTA) and stored at −20 °C until DNA extraction. The genomic DNA was extracted from 200 μL of EDTA-anticoagulated blood using a kit (PureLinkTM Genomic DNA Mini Kit, Invitrogen Corporation, Carlsbad, CA, USA), as directed by the manufacturer. The genomic DNA concentration was determined using spectrophotometry (NanoDrop^®^ ND- 2000 UV/Vis Spectrophotometer, Thermo Fisher Scientific Inc., Wilmington, DE, USA).

Between April and June 2019, blood samples were collected from 30 different foci in the Mediterranean region’s Alanya, Akseki, and Manavgat districts in Antalya, and Bozyazi and Anamur districts in Mersin (Figure 1). This region has a Mediterranean climate, with summers that are hot and humid and winters that are mild and rainy. Sheep and goats kept indoors in coastal villages during the winter are transported to the Taurus Mountains’ highlands at the beginning of spring and pastured there until autumn.

### 2.2. Nested PCR and RLB

Primers Nbab1F and Nbab1R [25] were used for the initial amplification of an approximately 1600 bp fragment of the *18S rRNA* gene in *Theileria* and *Babesia* to improve the quality of amplification. The hypervariable V4 region of the piroplasm *18S rRNA* gene was amplified by nested amplification using primers RLB-F2 and RLB-R2 [26] for the RLB assay. As previously described [27], PCR reactions were performed using PCR Sprint (Sensoquest, Göttingen, Germany).

RLB was conducted on the nested PCR product, as previously described [28]. Following amplification, 20 μL of all PCR products obtained from each DNA sample were diluted to a final volume of 150 μL with 2X SSPE/0.1% SDS buffer. For RLB hybridization, the samples were heated at 95–100 °C for 10 min in a Thermal Cycler and rapidly cooled on ice. The PCR products were then hybridized with probes specific to the genera and species of *Babesia* and *Theileria* that were linked to an RLB membrane. The primers and probes used in the study are listed in Supplementary Table S1.

### 2.3. Restriction Fragment Length Polymorphism (RFLP)

Due to the limitations of the RLB method in differentiating between *T. annulata* and *T. lestoquardi* species, the RFLP technique was used to identify these species [29,30,31,32]. In this study, RFLP analysis was performed on 12 DNA samples identified as *T. annulata*/*T. lestoquardi* by the RLB method. First, nested PCR (nPCR) was performed with TheiF1-TheiR1 and TheiF2-TheiR2 [29] primers, and the resulting nPCR amplicons were digested with HpaII restriction enzyme, according to the manufacturer’s protocol.

### 2.4. Sequencing and Phylogenetic Analyses

To confirm the *Theileria* and *Babesia* species detected as a result of the RLB analysis, the samples were subjected to sequence analysis. For the sequence analysis, an region of approximately 1400 bp of the *18S rRNA* gene was amplified using primers Nbab1F-Nbab1R and BT18F2-BT18R2 [25,33]. The obtained nucleotide sequences were compared to those in the NCBI database using BLAST analysis, and phylogenetic analysis was conducted using the Mega X program [34]. To compare the data from sheep and goats, Pearson’s chi-squared (2) test was used with SPSS version 15.00.

## 3. Results

The prevalence of various tick-borne parasites in the region was determined by analyzing the collected samples using molecular methods. Table 1 presents the frequency values of single- and mixed-piroplasm infections as a result of amplification products hybridized on the RLB membrane. PCR analysis revealed that 179 (28%) of 640 blood samples were infected with *Theileria* or *Babesia* species. Manavgat had the highest proportion of positive samples (48.2%), followed by Anamur (25.4%), Alanya (21.9%), and Akseki (18.2%). Bozyazi had the lowest prevalence of piroplasms (5.7%), and the difference between Manavgat and other provinces was statistically significant (*p* < 0.05).

PCR-based RLB revealed the presence of five species of piroplasms (*B. aktasi* n. sp., *B. ovis*, *Theileria* sp., *T. ovis*, and *T. annulata*). *Babesia aktasi* n. sp. was the most prevalent species in goats (22.5%) and was detected throughout each province. The frequencies of *B. ovis*, *Theileria* sp., *T. ovis*, and *T. annulata/lestoquardi* were 4%, 0.6%, 2.8%, and 2.4%, respectively. Although five different piroplasm species were detected in goats, only *T. ovis* was detected in sheep at a rate of 51.8% (Table 2).

**Figure 2:**
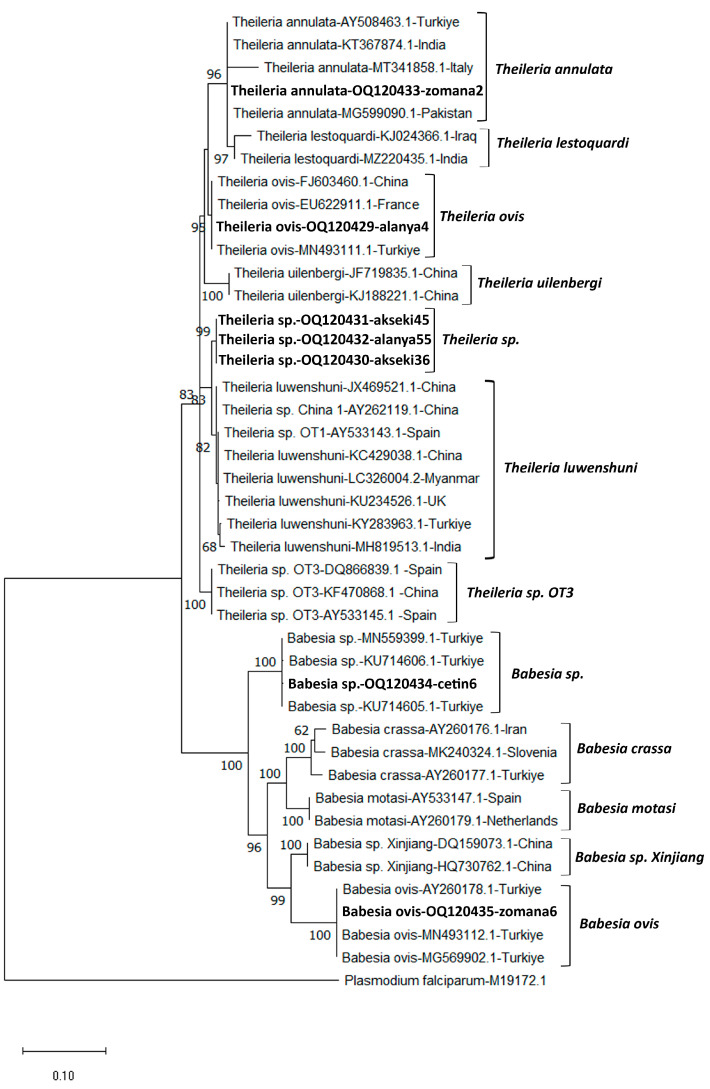
The phylogenetic tree generated using the Mega X program shows the phylogenetic relationship of *Theileria* and *Babesia* species (bold letters) identified in this study with other apicomplexan parasites. The evolutionary history was inferred based on the Tamura–Nei (G+I) model. Next to each branch is the percentage of replicate trees in which the associated taxa are clustered together in the bootstrap test (1000 replicates). Only bootstrap values higher than 50 are displayed next to the branches. To the right of each species name are the GenBank accession numbers. As an outgroup, *Plasmodium falciparum* (M19172) was utilized. The scale bar indicates the evolutionary distance in terms of nucleotide substitutions per site.

The RFLP analysis conducted to distinguish between *T. annulata* and *T. lestoquardi* species revealed that all 12 samples belonged to *T. annulata*. A 1400 bp region of the *18S rRNA* gene region of *B. aktasi* n. sp., *B. ovis*, *Theileria* sp., *T. ovis*, and *T. annulata* was sequenced and submitted to GenBank to confirm the RLB results (OQ120434, OQ120435, OQ120430-OQ120432, OQ120429, and OQ120433, respectively). The *Babesia aktasi* n. sp. sequence (OQ120434) showed a 99.6–99.8% sequence identity to *Babesia* sp. isolate oglak (MN559399.1), *Babesia* sp. MA-2016a isolate v5 (KU714605.1), *Babesia* sp. isolate Manay (OM864353.1), and *Babesia* sp. MA-2016a isolate v8 (KU714606.1) sequences isolated from goats in Türkiye. The sequence of *B. ovis* (OQ120435) showed a 99.6–99.9 sequence identity with existing sequences isolated from sheep (MN493112.1) and horses (MG569902.1) in Türkiye. *Theileria ovis* and *T. annulata* sequences were found to be 100% similar to the related sequences in GenBank. In this study, three samples (akseki36, akseki45, and 157 alanya55) were identified as *Theileria* sp., even though they reacted to the *T. luwenshuni*/OT1 probe in the RLB analysis. BLAST analysis revealed that the sequences (OQ120430-OQ120432) of these samples were 99.1% similar to *T. luwenshuni* isolates isolated from small ruminants in China (JX469514.1, JF719833.1, JF719832.1, AY262119.1). Although the nearly full sequence of the *18S rRNA* gene was similar to *T. luwenshuni*, nucleotide changes were observed in the hypervariable V4 region of this gene (Supplementary Table S2). In addition, phylogenetic analysis revealed that it formed a sister clade with *T. luwenshuni* sequences from various parts of the world (Figure 2). MEGA X was used to conduct phylogenetic analyses involving the DNA sequences identified in this study and *Theileria* and *Babeisa* species found in sheep and goats. Figure 2 depicts a tree of *18S rRNA* sequences based on the maximum probability and the Tamura–Nei model [34,35].

## 4. Discussion

Small ruminants have long been important components of rural life in Türkiye, particularly for farmers who live nomadic lifestyles [36]. Tick-borne diseases (TBDs) have a negative impact on small ruminants breeding in tropical and subtropical regions, including Türkiye, and cause significant economic losses to the livestock industry [37]. A new *Babesia* sp. (*B. aktasi* n. sp.) has been reported in goats using molecular methods in the Mediterranean region, where the nomadic lifestyle is prevalent [23]. Herein, we presented the molecular survey of *B. aktasi* n. sp. in sheep and goats from the Mediterranean region. RLB analysis revealed that 34.4% (220/640) of the samples collected from apparently healthy sheep and goats for this study reacted to specific probes (*T. ovis*, *Theileria* sp., *T. annulata*, *B. ovis*, and *B. aktasi* n. sp.). *Theileria ovis*, *T. luwenshuni*, *T. uilenbergi*, *Theileria* sp. OT1, *Theileria* sp. OT3, *Theileria* sp. MK, *B. ovis*, *B. motasi*, and *B. crassa* species were revealed in the molecular studies on blood parasites transmitted by ticks in sheep and goats in Türkiye [37]. 

A study involving 78 sheep and 122 goats in 2017 revealed that 5.7% (7/122) of the goats were infected with the new *Babesia* sp. This newly identified *Babesia* sp. was then isolated from splenectomized goats and characterized in terms of its genetic and morphological features [23]. According to the results of the present survey, a high frequency of *B. aktasi* n. sp. in goats was found (22.5%). This prevalence was observed to be quite high in comparison to the study that was conducted in 2017 [23], and the inclusion of samples from different locations (high-altitude areas) as well as the number of samples may have been effective in this regard. Furthermore, it is well known that the study area is a natural habitat for mountain goats. *Babesia aktasi* n. sp. is genetically similar to *B. odocoilei* and *B. venatorum* species that infect deer, rather than the *Babesia* species that infect sheep and goats [23,24]. In this study, sheep and goat samples were collected from high-altitude regions where they share the same habitat as mountain goats. Although we did not have any data on the presence of *B. aktasi* n. sp. in mountain goats, we speculated that they were hosts for this parasite. Despite its high prevalence in goats, the absence of *B. aktasi* n. sp. in sheep living in the same habitat may indicate host selectivity for the parasite. A previous study that supports our findings determined no *B. aktasi* n. sp. in sheep [23]. 

*Babesia ovis* is the main cause of small ruminant babesiosis, and it has a significant economic impact on the ovine industry in tropical and subtropical areas [1,5]. Ovine babesiosis caused by *B. ovis* has been reported serologically and molecularly in all geographical regions of Türkiye [37,38]. *Babesia ovis* seroprevalence was determined to be 49.2% by an IFA test and 29.9% by the rBoSA1-based enzyme-linked immunosorbent test (ELISA) in a nationwide seroepidemiological study on the subject [38]. Although there is no case registration system for ovine babesiosis, the molecular and serological data indicate that ovine babesiosis is a severe problem for Türkiye’s small ruminant industry. In this study, 4% of goats screened positive for *B. ovis*. Although the molecular prevalence is consistent with the results of previous studies [37,38,39,40], *B. ovis* was not detected in sheep.

Small ruminant theileriosis is not considered a major problem in Türkiye, and no outbreaks or cases of *T. lestoquardi*, *T. uilenbergi*, or *T. luwenshuni* species causing clinical infections have been reported [37]. *Theileria luwenshuni*/OT1 and *T. uilenbergi*, which have long been the cause of small ruminant theileriosis outbreaks with high morbidity and mortality rates for sheep and goats in various parts of China [41,42], have recently been reported molecularly in sheep and goats in Türkiye [39]. In addition, outbreaks of ovine theileriosis caused by *T. luwenshuni* infection have recently been identified in India and the United Kingdom through molecular detection [43,44]. The *18S rRNA* gene sequence of *Theileria* sp. OT1, isolated from sheep in Spain [19], revealed a 99.6% similarity to *T. luwenshuni*. Although the *18S rRNA* gene sequences are similar, there is no information on whether *Theileria* sp. OT1 is a different species. In fact, according to the *18S rRNA* gene sequence, *T. annulata*, the causative agent of tropical theileriosis and a serious infection in cattle, and *T. lestoquardi*, the cause of malignant ovine theileriosis, are 99–100% similar [31,45,46]. *Theileria luwenshuni*/OT1 was found in 2.6% of sheep in the only report published in Türkiye on the parasite [39]. In this study, in the RLB analysis, three goat samples were signaled against *T. luwenshuni*/OT1 probes, and these samples were considered *T. luwenshuni*/OT1, prior to the sequence analysis. The BLAST analysis of the *18S rRNA* gene region of these three samples revealed a 99.1% similarity to *T. luwenshuni* sequences isolated from small ruminants in China. However, when the nucleotide changes were analyzed, it was observed that the hypervariable V4 region contained the majority of these changes (Supplementary Figure S1). The *18S rRNA* gene’s hypervariable V4 region is crucial for elucidating the relationships between different isolates and species, and RLB probes are designed from this region [47]. In addition, it was named *Theileria* sp. in this study because it formed a sister clade with *T. luwenshuni* sequences isolated from different parts of the world (Figure 1). Surprisingly, *T. lestoquardi*, detected in neighboring countries [48,49], has never been documented in Türkiye to date. However, in this study, the prevalence of *T. annulata*, which is morphologically, biologically, and phylogenetically closely related to *T. lestoquardi*, was observed to be 2.4% in goats. These data are not surprising and support the previous data since *T. annulata* was reported in sheep and goats in several studies [30,31,32,50,51]. It has been reported that sheep can become infected with *T. annulata*, exhibit clinical signs of theileriosis, and develop immunity against *T. annulata* [52]. *Theileria ovis*, which is considered nonpathogenic, is a tick-borne protozoan agent that is most prevalent in sheep and goats in Türkiye. Epidemiological studies indicate that the prevalence of *T. ovis* in Türkiye varies from 9.2% to 68% in sheep and from 0.3% to 17.7% in goats [37,38]. In this study, the prevalence of *T. ovis* was determined to be 51.8% in sheep and 2.8% in goats, which is consistent with the previous study [37]. This difference between sheep and goats is influenced by seasonal activities and infection rates of vector tick species, animal infestation rates, distinct grazing behaviors of sheep and goats, and parasite epidemiology, according to the previous research [37,38]. Additionally, it is known that goats are more resistant to piroplasmosis than sheep. [17]. It is unknown, however, how long the *T. ovis* can remain in its host for adaptation in sheep and goats. Although epidemiological factors are believed to play a significant role in the difference in prevalence between *Theileria*/*Babesia* species in sheep and goats, host adaptation of these parasites is a significant factor that can affect prevalence.

## 5. Conclusions

In conclusion, we determined that *B. aktasi* n. sp. has a high prevalence in goats in Türkiye’s Mediterranean area. As a result of this result, we have preliminary evidence that this parasite presents good host adaptation for goats. In this study, we found *T. ovis*, *Theileira* sp., *T. annulata*, and *B. ovis* in goats and *T. ovis* in sheep. Furthermore, despite the fact that tick-borne pathogens are more common in sheep than in goats, *B. aktasi* n. sp. was only found in goats in this study. In order to solve the mysteries surrounding this parasite, detailed research on the parasite’s host adaptation and pathogenicity is expected in the future.

## Figures and Tables

**Table 1:** Prevalence of *Theileria* and *Babesia* species, including single and mixed infections, in different hosts and provinces using RLB.

			Single Infection (%)					Mixed Infection (%)			
Province	Host	n	*B. aktasi* n. sp.	*B. ovis*	*T. ovis*	*T. annulata*	*Theileria* sp.	*B. aktasi* n. sp. + *B. ovis*	*B. aktasi* n. sp. + *T. ovis*	*B. aktasi* n. sp. + *T. annulata*	*B. ovis* + *T. annulata*
Akseki	Sheep	9	-	-	8 (88.9%)	-	-	-	-	-	-
	Goat	131	11 (8.4%)	1 (0.8%)	-	1 (0.8%)	2 (1.5%)	-	2 (1.5%)	1 (0.8%)	-
	∑	26/140 (18.6%)	11 (7.9%)	1 (0.7%)	8 (5.7%)	1 (0.7%)	2 (1.4%)	-	2 (1.4%)	1 (0.7%)	-
Manavgat	Sheep	103	-	-	51 (58.3)	-	-	-	-	-	-
	Goat	204	66 (32.3%)	8 (3.9%)	-	2 (1%)	-	9 (4.4%)	11 (5.4%)	1 (0.5%)	-
	∑	148/307 (48.2%)	66 (21.5%)	8 (2.6%)	51 (16.6%)	2 (0.7%)	-	9 (2.9%)	11 (3.6%)	1 (0.3%)	-
Alanya	Sheep	9	-	-	8 (88.9%)	-	-	-	-	-	-
	Goat	55	3 (5.5%)	-	-	2 (3.6%)	1 (1.8%)	-	-	-	-
	∑	14/64 (21.9%)	3 (4.7%)	-	8 (12.5%)	2 (3.1%)	1 (1.6%)	-	-	-	-
Bozyazi	Sheep	-	-	-	-	-	-	-	-	-	-
	Goat	70	3 (4.3%)	-	-	1 (1.4%)	-	-	-	-	-
	∑	4/70 (5.7%)	3 (4.3%)	-	-	1 (1.4%)	-	-	-	-	-
Anamur	Sheep	16	-	-	4 (25%)	-	-	-	-	-	-
	Goat	43	6 (13.9%)	-	1 (2.3%)	2 (4.6%)	-	-	-	-	2 (4.6%)
	∑	15/59 (25.4%)	6 (10.2%)	-	5 (8.5%)	2 (3.4%)	-	-	-	-	2 (3.4%)
Total	Sheep	137	-	-	71 (51.8%)	-	-	-	-	-	-
	Goat	503	89 (17.7%)	9 (1.8%)	1 (0.2%)	8 (1.6%)	3 (0.6%)	9 (1.8%)	13 (2.6%)	2 (0.4%)	2 (0.4%)
	∑	207/640 (32.3%)	89 (13.9%)	9 (1.4%)	72 (11.3%)	8 (1.3%)	3 (0.5%)	9 (1.4%)	13 (2%)	2 (0.3%)	2 (0.3%)

**Table 2:** Frequencies (%) of tick-borne piroplasms (single and mixed infections) in sheep and goats detected by molecular tools (PCR and RLB) (n = 640).

		Identified Pathogens				
Host	No. Positive	*Babesia aktasi* n. sp.	*B. ovis*	*Theileria* sp.	*T. ovis*	*T. annulata*
Goat (n = 503)	89	+	-	-	-	-
	13	+	-	-	+	-
	9	-	+	-	-	-
	9	+	+	-	-	-
	8	-	-	-	-	+
	3	-	-	+	-	-
	2	+	-	-	-	+
	2	-	+	-	-	+
	1	-	-	-	+	-
∑	136/503 (27%)	113/503 (22.5%)	20/503 (4%)	3/503 (0.6%)	14/503 (2.8%)	12/503 (2.4%)
Sheep (n = 137)	71	-	-	-	+	-
∑	71/137 (51.8%)				71/137 (51.8%)	

## Data Availability

Data available in a publicly accessible repository.

## Supplementary Materials

The following supporting information can be downloaded at: https://www.mdpi.com/article/10.3390/pathogens12040514/s1, Figure S1: Alignment of the 18S rRNA V4 hypervariable region of *Theileria* sp. (bold) and *Theileria luwenshuni* genotypes. Nucleotide changes are indicated in a black box; Table S1: Primers and probes used in the study [53,54,55]; Table S2: Pairwise distance matrix comparing of the 18S rRNAV4 hypervariable region (~400 bp) of *Theileria* sp. (bold) to *Theileria luwenshuni* genotypes was created by Clustal2.1. Data represent % identity (*p*-distance).
