# Supplementary material for: Small Ruminant Piroplasmosis: High Prevalence of Babesia aktasi n. sp. in Goats in Türkiye
Source: Pathogens. 2023 Mar 26;12(4):514. doi: 10.3390/pathogens12040514 (PMC10142182; doi:10.3390/pathogens12040514)
Supplement: Supplementary file 1 [file pathogens-12-00514-s001.zip › pathogens-2268950-supplementary.pdf]

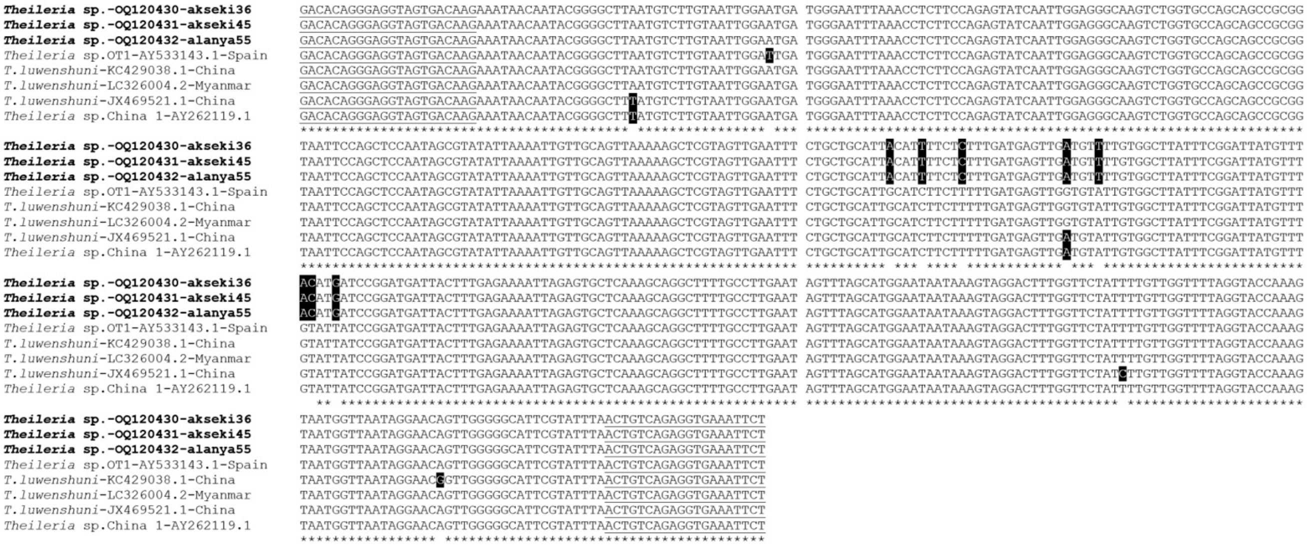

Supplemenatary Figure S1: Alignment of the 18S rRNA V4 hypervariable region of *Theileria* sp. (bold) and *Theileria luwenshuni* genotypes. Nucleotide changes are indicated on a black box.

Supplementary Table S1: Primers and probs used in the study.

Primers	Sequence (5′-3′)	References
Nbab-1F	AAGCCATGCATGTCTAAGTATAAGCTTTT	[25]
Nbab-1R	CTTCTCCTTCCTTTAAGTGATAAGGTTCAC	
RLB-F2	GACACAGGGAGGTAGTGACAAG	[26]
RLB-R2	biotin-CTAAGAATTTCACCTCTGACAGT	
**Probs**		
Catchall	AmMC6-TAATGGTTAATAGGA(AG)C(AG)GTTG	[19]
Theileria spp.	AmMC6-TGATGGGAATTTAAACC(CT)CTTCCA	[19]
T. ovis	AmMC6-TTTTGCTCCTTTACGAGTCTTTGC	[19]
T. separate	AmMC6- TCGGATGATACTTGTATTATC	[54]
T. lestoquardi	AmMC6-ATTGCTTGTGTCCCTCCG	[54]
T. uilenbergi	AmMC6-TGCATTTTCCGAGTGTTACT	[54]
T. luwenshuni	AmMC6-TCGGATGATACTTGTATTATC	[53]
Theileria sp. OT1	AmMC6-ATCTTCTTTTTGATGAGTTGGTGT	[19]
Theileria sp. OT3	AmMC6-ATTTTCTCTTTTTATATGAGTTTT	[19]
Theileria sp. MK	AmMC6-CATTGTTTCTTCTCATGTC	[20]
Babesia Catchall 1	AmMC6 -ATTAGAGTGTTTCAAGCAGAC	[55]
Babesia Catchall 2	AmMC6 -ACTAGAGTGTTTCAAACAGGC	[55]
B. ovis	AmMC6-GCGCGCGGCCTTTGCGTTTACT	[19]
B. motasi	AmMC6-ATTGGAGTATTGCGCTTGCTTTTT	[19]
B. crassa	AmMC6-TTATGGCCCGTTGGCTTAT	[19]
Babesia sp.	AmMC6-TGCCGTGAATCGACATTCGTC	[23]

AmMC6: Amino group

Supplemenatary Table S2: Pairwise distance matrix comparing of the 18S rRNAV4 hypervariable region (~400 bp) of *Theileria* sp. (bold) to *Theileria luwenshuni* genotypes was created by Clustal2.1. Data represent % identity (p-distance).

		**1**	**2**	**3**	**4**	**5**	**6**	**7**	**8**
**1**	***Theileria* sp.-OQ120430-akseki36**	100.00	100.00	100.00	97.21	97.21	97.44	97.21	97.44
**2**	***Theileria* sp.-OQ120431-akseki36**		100.00	100.00	97.21	97.21	97.44	97.21	97.44
**3**	***Theileria* sp.-OQ120432-alanya55**			100.00	97.21	97.21	97.44	97.21	97.44
**4**	*Theileria* sp.OT1-AY533143.1-Spain				100.00	99.53	99.77	99.07	99.30
**5**	*T.luwenshuni*-KC429038.1-China					100.00	99.77	99.07	99.30
**6**	*T.luwenshuni*-LC326004.2-Myanmar						100.00	99.30	99.53
**7**	*T.luwenshuni*-JX469521.1-China							100.00	99.77
**8**	*Theileria* sp.China 1-AY262119.1								100.00
